# Agnostic Biomarkers and Molecular Signatures in Colorectal Cancer—Guiding Chemotherapy and Predicting Response

**DOI:** 10.3390/biomedicines13082038

**Published:** 2025-08-21

**Authors:** Ilektra Kyrochristou, Georgios D. Lianos, Gerasimia D. Kyrochristou, Georgios Anagnostopoulos, Christina Bali, Stergios Boussios, Michail Mitsis, Dimitrios Schizas, Konstantinos Vlachos

**Affiliations:** 1 Department of Surgery, University Hospital of Ioannina, 45110 Ioannina, Greece; glianos@uoi.gr (G.D.L.);; 2 Department of Surgery, General Hospital of Nikaia and Piraeus, 18454 Athens, Greece; geor.anagnwstopoulos@gmail.com; 3 Faculty of Medicine, School of Health Sciences, University of Ioannina, 45110 Ioannina, Greece; 4 Department of Medical Oncology, University Hospital of Ioannina, 45500 Ioannina, Greece; 5 Faculty of Medicine, Health, and Social Care, Canterbury Christ Church University, Canterbury CT1 1QU, UK; 6 Faculty of Life Sciences & Medicine, School of Cancer & Pharmaceutical Sciences, King’s College London, Strand, London WC2R 2LS, UK; 7 Department of Research and Innovation, Medway NHS Foundation Trust, Gillingham Kent ME7 5NY, UK; 8 AELIA Organization, 57001 Thessaloniki, Greece; 9 Department of Surgery, National and Kapodistrian University of Athens, Laikon General Hospital, 11527 Athens, Greece; schizasad@gmail.com

**Keywords:** agnostic biomarkers, agnostic targets, colorectal cancer, molecular signatures in colorectal cancer, tumor-agnostic, chemotherapy

## Abstract

The concept of agnostic biomarkers—molecular modifications that guide therapy irrespective of tumor origin—has gained increasing relevance in oncology, including colorectal cancer (CRC). This review aims to critically evaluate the role of such biomarkers in CRC, highlighting their clinical significance as therapeutic targets and indicators of prognosis. Through a PubMed search using the terms “agnostic treatment AND colorectal cancer,” eight key studies were identified and qualitatively analyzed. We focus on several biomarkers commonly regarded as agnostic across tumor types, including *BRAF V600E* mutation, receptor tyrosine kinase (RTK) and *PI3K* fusions, the CpG island methylator phenotype (CIMP), high tumor mutational burden (TMB), and microsatellite instability (MSI). These markers are inspected for their prevalence in CRC, underlying pathophysiological mechanisms of cancer promotion, and predictive or prognostic implications. Moreover, we integrate findings from broader oncologic studies to contextualize the evolving role of agnostic biomarkers beyond organ-specific paradigms. Emerging evidence suggests that leveraging these molecular signatures may inform the use of targeted and immunotherapeutic agents as first-line options in select CRC populations. Collectively, agnostic biomarkers represent an auspicious avenue for personalizing CRC treatment, particularly in advanced-stage disease where traditional treatment options remain limited.

## 1. Introduction

As precision medicine continues to take the lead in modern healthcare, biomarker-driven cancer therapy has transformed the field of oncology. A deeper understanding of tumor biology and behavior has led to the identification and exploration of numerous agnostic targets, assessing their potential both as therapeutic drivers and as predictors of treatment response.

Agnostic biomarkers in colorectal cancer (CRC)—also referred to as tissue-agnostic or tumor-agnostic biomarkers—are molecular characteristics that can guide treatment decisions regardless of the cancer’s tissue or organ of origin [1]. These biomarkers encompass molecular signatures ranging from specific gene fusions to elevated tumor mutational burden (TMB), and they can be targeted with various chemotherapeutic and immunotherapeutic agents, irrespective of tumor site. The underlying concept is a “one-size-fits-many” approach, based on the presence of shared molecular aberrations across different solid tumors [2].

Since 2017, when pembrolizumab received U.S. Food and Drug Administration (FDA) approval as the first agnostic therapy for solid tumors—including colon cancer—significant advances have been made [3]. Developments in next-generation sequencing and the expansion of large genetic databases containing extensive molecular profiling data have enabled the recognition of additional potential biomarkers, such as *NTRK* fusions, *BRAF V600E* mutations, and *RET* fusions, alongside the development of corresponding targeted therapies [4,5,6,7].

This evolving therapeutic landscape offers new opportunities for organ-preserving treatment—capable of downstaging bulky, infiltrative, or metastatic tumors [8]—and for achieving complete or near-complete remission in early-stage disease through immunotherapy [9], ultimately improving survival outcomes and quality of life [10].

At present, six tissue-agnostic biomarkers have FDA-approved targeted or immune-based therapies for various cancers, including some forms of CRC. These include NTRK fusions, *BRAF V600E* mutations, *RET* fusions, *Her-2 positive* status, high tumor mutational burden (TMB-H), and deficient mismatch repair/high microsatellite instability (dMMR/MSI-H) [11].

Because these biomarkers are distributed across multiple tumor types, accumulating a substantial body of CRC-specific data remains challenging. This review aims to consolidate current knowledge on agnostic targets and biomarkers relevant to CRC treatment and prognosis. We present the most frequently reported molecular profiles in CRC, outline the agnostic targets recognized to date, and discuss biomarkers that have been associated with predicting response to neoadjuvant and systemic therapy.

## 2. Materials and Methods

Research was conducted in the PubMed database under the algorithm “agnostic treatment AND colorectal cancer”.

A total of 35 out of 130 articles were full-text examined. Finally, eight articles met the inclusion criteria mentioned below. The search took place in June 2025. Two independent researchers validated the results.

All original research articles involving sporadic colorectal cancer (CRC) patients who underwent molecular profiling and received tumor-agnostic treatment based on molecular characteristics were included. Studies were also eligible if they reported treatment response or clinical outcomes linked to specific molecular features of CRC, regardless of whether the intervention was explicitly labeled “tumor-agnostic.” Studies including a broader population (patients with various types of tumors, not only CRC), were included in the synthesis, only if the subgroup of CRC patients was clearly defined, as well as their characteristics and results separately reported from the rest of solid tumors.

Articles were excluded if they:

- Included multiple tumor types without providing a clearly differentiated analysis for CRC patients;
- Lacked clinical implications of the molecular findings (e.g., purely mechanistic or preclinical studies without patient outcome data);
- Were review articles, editorials, or conference abstracts; or
- Were not published in English.

All research protocols were evaluated for their risk of bias using the ROBINS-I-V2 tool (Table 1) [12,13,14,15,16,17,18,19]. Results were presented descriptively.

## 3. Results

Overall, eight articles were included in the qualitative synthesis, including a total of 16,330 CRC patients of various ages; most were older than 55 years old, but due to heterogeneity in the manner of presentation, a synthesis of the results was not possible [12,13,14,15,16,17,18]. Five studies provided information on the patients’ sex (total N of patients = 1480), with 46% being males [12,14,15,17,18]. Six studies provided information on the cancer site, with 3377 out of 16,233 cancers being in the rectum. Demographic information of the patients is presented in Table 2.

Regarding the samples tested in each case for molecular profiling, four researchers used tissue biopsies, three used venous blood/plasma, and one used both. Loree et al. [15] tested both plasma and tissue to compare the TMB on the two and reported that plasma TMB was significantly higher than tissue’s [median, 15.3 mutations/Mb (IQR, 9.5–26.2) vs. 6.5 mutations/Mb (3.9–12.0), *p* = 8.7 × 10^−6^)] [15]. When the clonal TMB was used, this difference was smaller, but the correlation between the plasma and the tissue TMB remained weak. The researchers continued to find a cut-off value of plasma TMB that would influence overall survival (OS), which was calculated as >10.6 mutations/Mb [HR, 0.20 (95% CI, 0.068–0.59); *p* = 0.0035].

Most performed next-generation sequencing with commercially available systems or reported results from databases that used such systems. microRNA (miRNA) and cell-free DNA (cfDNA) were also detected with immunochemistry methods in some instances. Information on the samples’ origin and testing is presented in Table 3.

### 3.1. Most Frequently Altered Genes and Pathways in Sporadic CRC–Agnostic Targets

All researchers reported the genomic profiles and the most frequently altered genes and pathways in their patient cohorts. Alterations in APC, KRAS, BRAF V600E, RTK, and *PI3K* fusions were the most frequently described. C- and N-terminal mutations of *APC* were not categorized separately. In an extended molecular profiling study, Chatila et al. reported that the most altered genes and pathways were *APC*, *TP53*, *KRAS*, *FBXW7*, *PIK3CA*, and WNT [12]. Arter et al. identified *NTRK*-positive and *RET*-positive CRC as distinct disease entities that could potentially respond to immunotherapy with agents such as pembrolizumab, dostarlimab, or nivolumab in combination with ipilimumab [16]. They also reported that *NTRK* fusions are typically mutually exclusive with activating mutations in the *RAS* and *BRAF* genes [12,16].

In a large cohort of 14,821 CRC patients, 153 unique RTK fusions were identified, the most common being *FGFR1*, *EGFR*, *ERBB2*, *NTRK1*, *RET*, *FGFR2*, *FLT1*, *FLT3*, *FLT4*, and *ALK* [16]. The CRC group included both partner fusions (promoter-driven) and intragenic rearrangements, the latter most often resulting in loss-of-function mutations.

Loree et al. reported a high prevalence of mutated DNA repair genes in patients with metastatic CRC, with *BRCA2* and *ATM* alterations present in 12% and 14% of their study population, respectively [15]. These mutations were more effectively detected when sequencing was performed on DNA extracted from tumor tissue, whereas plasma samples showed lower detection rates.

Gouda et al. suggested that CRC patients may have higher levels of CpG motifs in their plasma cfDNA [13]. In their cohort, 85% of patients demonstrated methylated cfDNA, regardless of *KRAS* status. However, the study included only 20 individuals, underscoring the need for further validation.

Bartlett et al. examined the role of miRNAs in pathway alterations in CRC and concluded that miR-155 and miR-22 significantly influenced these pathways (*p* = 0.0001) and TGF-β signaling (*p* = 0.008) [18]. Three of the twenty-one genes predicted to be affected by these miRNAs—*SMAD2*, *SMAD4*, and *TGFBR2*—were shared between pathways related to colorectal adenocarcinoma (COAD) and TGF-β signaling.

A summary of the most frequently altered genes and pathways is provided in Table 4.

### 3.2. Correlation Between the MMR-MSI Status and Distinct Gene Fusions

Several gene fusions have been linked to specific MSI statuses. Chatila et al. reported that dMMR/MSI-H (deficient mismatch repair/microsatellite instability–high) tumors had fewer *TP53* mutations (38% vs. 81%, *p* < 0.001), *APC* mutations (51% vs. 81%), and whole-genome duplication (WGD) events (0% vs. 40.1%, *p* = 0.026) compared to other tumors [12]. Oncogenic *KRAS* alterations were detected in 42% of proficient MMR (pMMR)/microsatellite stable (MSS) tumors.

In the study by Arter et al., MSI status was available for 1482 patients [16]. Notably, all *NTRK-positive* CRCs (100%) were dMMR. Among patients with *BRAF V600E* mutations, 38% were dMMR/MSI-H. KRAS G12D mutations were associated with dMMR/MSI-H in 7.4% of cases, whereas *KRAS G12C* and *KRAS G12A* mutations were exclusively found in MSS tumors (100%) [16]. The MSI-H tumors had a significantly higher tumor mutational burden (TMB) (mean 56.1 ± 19.7, median 44) compared to MSS tumors (mean 11 ± 18.4, median 6). *NTRK-positive* CRCs have also been shown to exhibit higher TMB and are more frequently associated with MSI-H status [16].

Regarding *RET* rearrangements, Pietrantonio et al. found that RET-negative tumors were MSS in 93% of cases [17]. RET-rearranged tumors were almost evenly distributed between MSI-H (48%) and MSS (52%). Interestingly, in this cohort, six patients with MSI-H, RAS/BRAF wild-type, right-sided tumors had a 23-fold higher likelihood of harboring RET rearrangements.

*NTRK* fusions are more often detected in dMMR/MSI-H CRC with MLH1 promoter hypermethylation and wild-type *RAS* and *BRAF*, with reported rates ranging from 17% to 44% [20,21]. MSI status is summarized in Table 4.

### 3.3. Agnostic Biomarkers and Pathological Characteristics of CRC

Arter et al. reported that *RET* fusions in CRC define a distinct subgroup of patients who may benefit from targeted therapy [16]. They observed a higher prevalence of *RET* mutations in patients over 66 years old and found equal proportions of MSS and MSI-H tumors in this group.

Conversely, Pietrantonio et al. reported that *RET-mutated* CRCs were more often MSI-H and located in the right colon (55% right-sided vs. 32% left-sided, *p* = 0.013) [17]. They also suggested that patients with metastatic CRC harboring a *RET* rearrangement may have poorer overall survival (OS) and could benefit from RET inhibitors.

In the literature, BRAF-mutated CRCs are frequently associated with serrated adenomas, particularly in the ascending colon, and occur more often in women and older patients (>60 years). Furthermore, *BRAF V600E*-mutated CRCs are often poorly differentiated and of mucinous histotype [22].

### 3.4. Agnostic Biomarkers of Oncological Outcomes and Response to CRC Treatments

Six of the studies provided data regarding the oncological outcomes and the response either to neoadjuvant treatment or adjuvant chemotherapy/immunotherapy (Table 5).

First, Chatila et al. reported that in the multivariate logistic regression, no significant associations between clinical variables, genomic variables, and complete response (CR) were noted [12]. However, a borderline significance of the relation between the *KRAS* mutations and poorer disease-free survival (DFS) was demonstrated (*p* = 0.04). At a transcriptomic level, the carbonic anhydrase 9 (CA9) had a prognostic value in pMMR/MSS tumors with mutated *KRAS* or *PIK3CA*, particularly in the double-mutants. Eight genes were overexpressed in the incomplete response group of patients, among others, the insulin-like growth factor 2 (IGF2) and L1 cell adhesion molecule (L1CAM).

Since we know that dMMR/MSI tumors have a distinct immunologic profile, Chatila et al. studied this subgroup to find a set of immune hot tumors that they named IG3 [12]. These tumors demonstrated extensive immune infiltration, less frequent *APC* and *TP53* mutations, better response to neoadjuvant treatment, and DFS.

Gouda et al. studied a population of CRC patients all with KRAS-mutated tumors, pointing out a relation between the *KRAS (+) tumors* with high methylated ctDNA amounts and a shorter median progression-free survival (PFS) [13]. This observation suggests that even in groups with this molecular signature, further categorization of the patients is possible and would help in better patient selection for future studies. In fact, for stage IV patients with *KRAS*-mutated tumors, Gouda reported that methylated ctDNA was associated with a median PFS of 8 weeks [95% CI, 4.3–11.7] versus 54 weeks [95% CI: 0–122.8] for the low methylated ctDNA group (*p* = 0.027).

In terms of response to neoadjuvant treatment in this group of patients with metastatic CRC, 180 patients of Loree et al. received a combination of durvalumab and tremelimumab as precision treatment for Programmed Death-Ligand 1 (PD-L1)- and Cytotoxic T-Lymphocyte-Associated Protein 4 (CTLA4)-positive tumors [15]. They achieved an improvement in median OS by 2.50 months compared with best supportive care [HR, 0.72 (90% CI, 0.54–0.97); *p* = 0.07]. No significant difference in PFS was reported.

As reported by Arter et al., TMB-H is associated with MSI tumors, also indicating a better response to immunotherapy [16]. According to the CheckMate 142 trial [23], out of 74 patients with dMMR/MSI-H mCRC who have received at least three prior therapies, nivolumab achieved a response rate of 68.9% (95% CI 57.1–79.2%), with patients demonstrating disease control for ≥12 weeks.

Some researchers imply that instead of the TMB, tumor neoantigen burden (TNB) might be a significant prognostic and predictive factor in CRC [24]. TNB is defined as the number of neoantigens per megabase in the genome region and is supposedly a better biomarker than TMB when it comes to immunotherapy response. Larger-scale studies are needed to clarify the significance of these markers, especially in CRC.

In CRC patients, a TMB-H indicates the increased production of neoantigens, which are recognized and attacked by the immune system. This results in augmented tumor immunogenicity, leading to better response in immunotherapy of certain tumors. Although the role of TMB as a prognostic biomarker of response to treatment remains controversial, since not all mutations result in the production of neoantigens, there is increasing evidence of its significance [24].

Pietrantonio et al. pointed out that tumors with *RET* mutated genes (e.g., CCDC6-RET) tend to be unrecognized as tumors with a distinct molecular signature, although they constantly demonstrated a worse OS, reporting in particular a median OS 14.0 versus 38.0 months of the *RET negative* CRC patients (HR, 4.59; 95% CI, 3.64–32.66; *p* < 0.001) [17]. They recognized the two most frequent fusions, *NCOA4-RET* and *CCDC6-RET*, mainly in older patients (median age was 66 years), and right-sided tumors (55% versus 32%, *p* = 0.013). Moreover, they reported that even for patients treated with RXDX105 (a multi-kinase inhibitor with potent activity against *RET*), the negative impact on prognosis remained significant after treatment for this group of patients (10.0 versus 38.0 months, HR, 4.57; 95% CI, 3.48–32.64; *p* < 0.001). Primary tumor location, *RAS* and *BRAF* mutations, and MSI status were not associated with worse OS.

Lastly, in the still ongoing study of Piha-Paul et al., patients with solid tumors characterized by PI3K pathway alterations were treated with buparlisib after progression of their disease despite other conventional treatments [19]. The idea was to select candidates that respond better to immunotherapy, as the PI3K pathway is linked to enhanced PD-L1 expression and higher TMB. But the results are not encouraging so far for CRC patients, who demonstrate no clinical benefit, implying that larger-scale studies are needed to validate or overturn these preliminary outcomes.

## 4. Discussion

Colorectal cancer (CRC) treatment has been profoundly transformed by the integration of precision medicine into surgical and oncologic care. The use of advanced technologies and molecular biomarkers now enables more personalized and effective treatment planning.

Agnostic targets are defined as distinct molecular features of cancer: genomic biomarkers such as mutations, gene fusions or rearrangements, tumor mutational burden (TMB), and microsatellite instability (MSI). Agnostic treatment approaches target a shared molecular alteration across different cancer types, regardless of the tumor’s tissue of origin [6,7].

The concept of tumor-agnostic therapy gained significant attention in 2017, when the U.S. Food and Drug Administration (FDA) approved pembrolizumab as the first tumor-agnostic drug for the treatment of solid cancers [5]. Since then, five additional agnostic treatments have been approved for CRC: dabrafenib, trametinib, selpercatinib, larotrectinib, and entrectinib [11].

In CRC, several molecular targets and biomarkers are considered in the selection of agnostic therapies and in overall treatment planning. These include *BRAF V600E* mutations, *NTRK fusions*, *RET fusions*, *KRAS mutations*, *PIK3CA mutations*, TMB-high (TMB-H), and deficient mismatch repair/high microsatellite instability (dMMR/MSI-H).

### 4.1. Emerging Agnostic Biomarkers

#### 4.1.1. BRAF V600E Mutations

*BRAF* mutations are detected in 5–15% of CRC patients [24]. Three distinct mutations of the *BRAF* gene have been acknowledged. Class I mutation refers to the *BRAF* V600E mutation and is the most common, found in 8–12% of patients with metastatic CRC (mCRC) have this mutation [25,26]. Class II *BRAF* mutations are found in 13% of CRC patients and concern the K601E/N/K, the T599K/I, or the fusion of the BRAF kinase domain. Finally, Class III BRAF alterations result in the formation of heterodimers, whose interaction with RAS or CRAF amplifies the effects of upstream signals and tumorigenesis [27].

*BRAF* V600E mutation results in an elevation in BRAF kinase activity, spanning from 130 to 700 times that of the wild-type, leading to prolonged aberrant activation of the Mitogen-activated Protein Kinase (MAPK) pathway and driving the initiation and progression of cancer [27]. But how are all these features translated into clinical data?

Regarding its prognostic significance, it has been reported that patients with CRC stage II and III have worse OS, but no difference in their DFS from patients without the mutation [28]. In a retrospective analysis of three trials, Roth et al. also concluded that *BRAF* mutations had a prognostic significance concerning OS but did not affect the possibility of relapse [29]. In the same analysis of almost 1500 cell blocks, a reverse relationship between the *KRAS* and the *BRAF* mutation was found.

In 2019, Loupakis et al. developed an intriguing prognostic score for *BRAF*-mutated mCRC, including eight covariates: Eastern Cooperative Oncology Group (ECOG) Performance Status, CA19.9, Lactate Dehydrogenase (LDH), NLR (Neutrophil-to-Lymphocyte Ratio), tumor grading, liver metastases, lung metastases, and nodal metastases [30]. A complete score could be any figure between 0 and 16. Referring to the score analysis, three different risk categories were defined—low (0–4), intermediate (5–8), and high (≥9). The median OS for patients included in the high-risk group was 6.6 months, for the intermediate-risk group, it was 15.5 months, and for the low-risk group, it was 29.6 months. These results indicate that among *BRAF*-mutated mCRC patients, distinct groups of different prognoses exist, a fact that could alter decision-making.

Today, there are several BRAF inhibitors used as targeted therapy regimens in the treatment of CRC. Dabrafenib has been FDA-approved. In research of Salama et al., including patients suffering from melanoma, CRC, and thyroid cancer, the confirmed objective response rate (ORR) was 38% (90% CI, 22.9–54.9%) with *p* < 0.0001 against a null rate of 5%, and PFS was 11.4 months [31]. Another inhibitor, vemurafenib, has been tested in non-melanoma patients with 13 different types of solid tumors (among them CRC), in the VE-BASKET study. Patients of this cohort demonstrated a response rate of 33%, a median PFS of 5.8 months, and a median OS of 17.6 months [32]. These results are encouraging for CRC patients, but due to the small number of *BRAF*-mutated CRC cancers diagnosed, larger-scale studies are needed to validate the results.

Nonetheless, the first-line treatment for *BRAF*-mutated mCRC patients remains the combination of chemotherapy and anti-EGFR/VEGF biological therapy. But keeping in mind that in the context of clinical studies, this combination achieves an ORR of 41% and PFS of 6.4–10.9 months, the introduction of a new treatment regimen would be crucial for better treatment outcomes [33].

#### 4.1.2. NTRK Fusions

NTRK fusions constitute targetable mutations occurring in various types of cancer. Even though in CRC patients their prevalence ranges from 0.35% to 0.7%, a brief reference will be made here, as this distinct group of NTRK mut (+) CRC patients may benefit from specific therapeutic regimens [5].

*NTRK* genes (*NTRK1/2/3*) encode tropomyosin receptor kinase (trk) proteins (TrkA/B/C). When mutated and fused with other genes, these genes result in a non-stop activity of the TRK proteins, leading to uncontrolled proliferation of cells, tumorigenesis, and cancer progression [34].

The data on their prognostic significance are doubtful, with some claiming that they are associated with a worse prognosis, OS, and DFS, especially those with MSI-H and RAS/BRAF wild-type status, while others demonstrate that there is no significant prognostic value [35,36].

Several clinical trials, such as the LOXO-TRK-14001, SCOUT, and NAVIGATE, have taken place to explore the potential benefit of a TRK inhibitor like larotrectinib in patients with NTRK fusion-positive tumors, including those with CRC [37,38,39]. In these studies, a total of twelve cancer types were investigated, but results on CRC patients were limited in number.

#### 4.1.3. RET Fusions

The *RET gene* is a proto-oncogene that encodes a receptor tyrosine kinase, whose most frequent alteration results in the lack of the transmembrane domain, leading to the production of a chimeric protein that continuously activates the RET signaling pathway and leads to uncontrolled cell proliferation, growth, and migration [40].

However, the correlation between the RET fusions and CRC is still open to interpretation, as other investigators suggest that enhanced methylation, and therefore downregulation of the *RET* proto-oncogene, is present in CRC patients, suggesting a tumor suppressive function rather than a promoter gene activity [41]. In addition, this suppressive function of RET was supported by Ashkboos et al., who, via immunohistochemical analyses, demonstrated a reduction in RET protein expression in CRC tissue when compared to adjacent normal tissue [42].

Despite this conflicting data, *RET* fusions are considered an “actionable” alteration. Targeted therapies, such as selpercatinib and pralsetinib, are used with great success in other types of RET-mutated cancers and hold promise to enhance the CRC treatment options [43]. According to the European Society for Medical Oncology (ESMO), testing for *RET fusions* is recommended for patients with metastatic CRC who have right-sided, MSI-high, and *RAS/BRAF wild-type* tumors, as they would probably benefit from precision medicine regimens as mentioned above [44]. However, the results of ongoing clinical trials are needed to further justify this suggestion.

#### 4.1.4. KRAS Mutations

*KRAS* mutations are one of the most studied groups of molecular signatures and targets in CRC patients, as they are extremely often (42% of CRC patients have *KRAS* mutations) [45]. When mutated, the GTPase KRAS protein disrupts the hydrolysis of GTP and/or enhances nucleotide exchange, contributing to a continuous activation of downstream signaling pathways and promoting cell proliferation.

In terms of tumor aggressiveness, *KRAS* mutations are associated with more aggressive tumor behavior and a higher risk of metastasis, especially liver lesions [45,46]. Moreover, they were associated with poorer OS and PFS, with the median PFS being reported around 28 months (95% CI, 22.206–33.794 months) for KRAS wild-type patients, while for patients with *KRAS* mutations, the PFS is reported about 19 months (95% CI, 15.678–22.322 months) [47,48,49,50].

Anti-EGFR (epidermal growth factor receptor) treatments, such as panitumumab or cetuximab monotherapy, or cetuximab in combination with irinotecan or other chemotherapeutic agents, have been tested for their efficacy against *KRAS*-mutated CRC, with devastating results [51,52,53]. Indicatively, De Roock et al. reported a median OS of the mCRC *KRAS wild-type* group of approximately 43 weeks on average, versus 27.3 weeks on the mutant group (*p* = 0.020) [51]. Beyond that, there are references to higher percentages of stable disease or progression to standard chemotherapy in patients with *KRAS*-mutated colorectal tumors (81% versus 62% for the KRAS wild-type group) [46].

The mutated *KRAS* was historically considered undruggable due to its lack of a clear binding pocket for small-molecule inhibitors. However, sotorasib and adagrasib are examples of KRASG12C inhibitors showing promise in clinical trials [52]. Modern research also explores other strategies, like targeting the cysteine in the *KRAS G12C* mutation or using G4-ligand compounds to target KRAS [54,55].

#### 4.1.5. PIK3CA Gene Mutations

*PIK3CA* gene mutation affects approximately 15–20% of CRC patients, leading to the persistent activation of the PI3K/AKT/mTOR signaling pathway, and thus promoting proliferation, invasion, metastasis, and drug resistance [56]. They are more often detected in male patients, with right-sided colon cancers, and at advanced stages at diagnosis.

Currently, there are limited data relating these mutations to OS or DFS [19,57]. However, researchers quote that *PIK3CA* mutations can influence the effectiveness of certain chemotherapeutic drugs. More specifically, CRC patients with *PIK3CA* mutations may exhibit a reduced response to anti-angiogenic drugs, such as bevacizumab [58]. Conversely, they may be detected in CRC patients whose tumor cells are more sensitive to mechanistic Target of Rapamycin (mTOR) inhibitors, such as everolimus and rapamycin; however, these interactions have only been studied at a clinical trial level [57,59].

### 4.2. Established Agnostic Biomarkers in CRC

#### 4.2.1. High Tumor Mutation Burden (TMB)

TMB is defined as the number of mutations found in the DNA of cancer cells. As a biomarker, it predicts how efficient certain types of immunotherapies, like checkpoint inhibitors, might be against various types of cancer [60]. In CRC, a TMB-H is generally defined as a tumor with more than 10 mutations per megabase (mut/Mb) of tumor DNA.

Wang et al. presented a series of patients with *KRAS*-mutated CRC tumors [60]. Using a cut-off value of 10 mut/Mb, they concluded that a TMB-H was an independent indicator of better prognosis, probably due to the higher response rate of this group of patients to immunotherapy. This result was not repeated when *APC*-mutated CRC patients were also included in the analysis.

But conflicting data regarding the TMB-H came from another significant observation, made by Huang et al. [61,62]. They observed a positive correlation between the prevalence of methylated positions (*POU3F3*, *SYN2*, and *TMEM178A* genes) and TMB-H (*p* = 0.45) and reported a worse prognosis for patients having the combination of methylated genes and TMB-H.

#### 4.2.2. Deficient Mismatch Repair/High Microsatellite Instability (dMMR/MSI-H)

A tumor’s inability to properly repair DNA errors and a resulting high degree of genetic mutations is described by the terms dMMR and MSI-H. dMMR/MSI-H is a pan-tumor phenotype found in nearly 15% of all CRCs [63]. Immune cell Programmed Death-Ligand 1 (PD-L1) expression is significantly higher in MSI-H CRC compared to pMMR (MSI-L) tumors, with no notable differences observed among the various MSI-H molecular subtypes. The recommended screening methods for detecting MMR deficiency include immunohistochemistry and/or MSI testing. However, translating the biological and technical heterogeneity of MSI assessment into clinically actionable data remains challenging. Literature reports indicate that immunohistochemical evaluation of MMR proteins can yield variable results for the same germline mutation, a phenomenon potentially attributable to accompanying somatic mutations [64].

These tumors demonstrate a distinct pathological profile, being mostly right-sided primary, mucinous, and poorly differentiated tumors, which often present BRAF mutations [65]. Approximately 20% of stage II, 12% of stage III, and 4% of stage IV CRC tumors are characterized as dMMR/MSI-H, suggesting a positive correlation between the dMMR/MSI-H status and earlier stages of CRC, along with a better prognosis [65,66].

The 2015 KEYNOTE-016 trial evaluated the clinical efficacy of Pembrolizumab in patients with pMMR/MSS mCRC, dMMR/MSI-H mCRC, and dMMR/MSI-H non-CRC [67]. In this study, the immune-related objective response rate and immune-related PFS rate were 40% and 78% respectively, for dMMR/MSI-H mCRC and 0% (0 of 18 patients) and 11% for pMMR/MSS mCRC. Since then, other researchers have also proved the significance of the MSI status in the response to immunotherapy, resulting in the approval by the FDA of pembrolizumab alone as a first-line treatment for patients with MSI-H/dMMR CRC that is unresectable or metastatic [68]. This addition has made it clear that from now on, immunotherapy is now prioritized over traditional chemotherapy ± targeted therapy.

All the above data highlight the transformative potential of genomic profiling and the application of precision medicine strategies in the treatment of CRC. Agnostic biomarkers constitute the guidance to variable innovative treatment patterns, conceptualized in the context of unique genetic landscapes of individual tumors and rare cancer subtypes.

### 4.3. Limitations of Tumor-Agnostic Biomarker Applications in CRC

While tumor-agnostic biomarkers offer a promising trail toward personalized medicine in CRC treatment, several limitations hinder their broad clinical utility. One major concern is the lack of standardization in molecular testing. Diagnostic platforms vary in terms of gene panel content, sequencing methods, and bioinformatics pipelines, resulting in inconsistencies in biomarker detection and interpretation, and reproducibility of findings [69]. Moreover, not all tests are validated for clinical use, which can compromise decision-making in real-world settings.

Another critical limitation is the development of resistance mechanisms. Tumor heterogeneity and adaptive resistance—such as bypass signaling in *BRAF*(+) CRC or secondary fusions—underscore the complexity of interpreting agnostic biomarkers into durable treatment tactics [70]. These challenges highlight the need for ongoing molecular monitoring and combination therapies tailored to evolving tumor profiles.

Finally, disparities in access to comprehensive genomic profiling limit the real-world impact of agnostic approaches. High costs, insurance restrictions, and uneven availability of testing infrastructure contribute to underutilization, especially in low-resource settings, jeopardizing the predictive accuracy and generalizability of agnostic biomarkers across diverse patient assemblies [71]. Without addressing these systemic barriers, the benefits of precision oncology risk being confined to a narrow segment of the CRC population.

The current review exhibits some limitations, in terms of lack of a multi-base search and the conduction of systematic results. Moreover, due to the limited data in the literature, our observations need to be validated from future larger-scale metanalysis.

## 5. Conclusions

The advent of agnostic biomarkers has redefined the therapeutic landscape of CRC, enabling treatment selection based on molecular signatures rather than tissue origins. Biomarkers such as BRAF V600E mutations, NTRK and RET fusions, high tumor mutational burden (TMB-H), and dMMR/MSI-H have not only expanded the armamentarium of targeted and immunotherapeutic agents but have also facilitated more personalized care with the potential for improved survival and quality of life.

Clinical implications include earlier identification of eligible patients through comprehensive genomic profiling, and the adoption of biomarker-driven therapies in both metastatic and, increasingly, neoadjuvant workflows. The growing evidence suggests that tumor-agnostic approaches may achieve meaningful tumor downstaging, durable responses, and better tolerability compared with conventional regimens, particularly in biomarker-enriched subgroups.

Despite these advances, knowledge gaps persist. Limited data for several agnostic biomarkers due to their low prevalence, high heterogeneity in testing methods, incomplete comprehension of resistance mechanisms, and the lack of validated predictive biomarkers for certain agents further limit clinical applicability.

Future research directions should focus on large-scale, multi-institutional studies to better define the prognostic and predictive value of rare agnostic biomarkers in CRC, elucidate mechanisms of primary and acquired resistance, and optimize sequencing or combination strategies with current modalities. Longitudinal studies should be the priority of research to assess long-term survival, quality-of-life outcomes, and cost-effectiveness of agnostic therapies. Finally, expanding access to comprehensive genomic profiling and integrating liquid biopsy approaches could accelerate the identification of eligible patients and enable real-time treatment adaptation.

In summary, agnostic biomarkers represent a paradigm shift in CRC management, bridging molecular oncology and precision therapeutics. Taking advantage of their full potential will require a combination of robust clinical evidence, equitable access to testing, and continued innovation in targeted drug development.

## Figures and Tables

**Table 1:** Bias assessment of studies according to the ROBINS-I-V2 tool.

Researcher	Domain 1 *	Domain 2 *	Domain 3 *	Domain 4 *	Domain 5 *
Chatila et al. [10]	Low concern	Low concern	Low concern	Low concern	Low concern
Gouda et al. [11]	Low concern	Low concern	Low concern	Low concern	Low concern
Zhu et al. [12]	Low concern	Some concerns	Some concerns	Low concern	Low concern
Loree et al. [13]	Low concern	Low concern	Low concern	Low concern	Low concern
Arter et al. [14]	Low concern	Some concerns	Some concerns	Low concern	Some concerns
Pietrantonio et al. [15]	Low concern	Low concern	Low concern	Low concern	Low concern
Bartlett et al. [16]	Low concern	Low concern	Some concerns	Low concern	Low concern
Piha-Paul et al. [17]	Low concern	Low concern	Some concerns	Low concern	Low concern

* D1: confounding, D2: classification of intervention, D3: selection into study, D4: deviations from intended intervention, D5: missing data.

**Table 2:** Patient demographics.

Researcher	Main Research Goal	No. of Patients (Male/Female)	Age (Years)	Tumor Site
Chatila et al. [10]	Recognition of tumor-agnostic biomarkers of response to neoadjuvant chemotherapy	692 (301/391)	<50	Rectum (100%)
Gouda et al. [11]	Test of methylation-specific signatures as mutation-agnostic biomarkers	20	50 (mean)	
Zhu et al. [12]	Recognition of cell-free methylated DNA markers as agnostic biomarkers of prognosis and treatment response	35 (19/16)	<55	Left colon (60%) Right colon (40%)
Loree et al. [13]	Comparison of plasma to tissue TMB * as an agnostic biomarker of response to chemotherapy	180 (59/121)	<65 y (n = 93, 51.7%) >65 y (n = 87, 48.3%)	Rectum (15%) Left colon (56%) Right colon (27%) Unknown (2%)
Arter et al. [14]	Recognition of *NTRK* and *RET* fusions as potential agnostic targets of therapy in CRC	14,812	63.5 (mean)	Colon (83%) Rectum (17%)
Pietrantonio et al. [15]	Recognition of *RET* fusions as agnostic targets and biomarkers in CRC	24 (10/14)	66 (median)	Right colon (54%) Left colon (45%)
Bartlett et al. [16]	Recognition of TGF-β as an agnostic target of immunotherapy	549 (292/257)	64.5 (mean)	Right colon (36%) Left colon (38%) Rectum (26%)
Piha-Paul et al. [17]	Test of buparlisib as an efficient and safe drug targeting *PI3K* activated pathway in the therapy of CRC	18	NP	NP

* Abbreviations: TMB: tumor mutation burden, NTRK: neurotrophic tyrosine receptor kinase gene, RET: Rearranged during Transfection gene, CRC: colorectal cancer, TGF-β: Transforming Growth Factor beta, PI3K pathway: Phosphatidylinositol 3-kinase pathway, NP: not provided.

**Table 3:** Sample types and molecular profiling methods.

Researcher	No. of Patients	Sample Type	Sequence Desired to Be Addressed	Profiling Method
Chatila et al. [10]	692	Tissue biopsies	Gene fusions	MSK-IMPACT sequencing
Gouda et al. [11]	20	Venous blood	Methylated CpG areas	NextSeq 500 MID Output Flow cell
Zhu et al. [12]	35	Venous blood	Methylated cfDNA	Bisulfite-converted DNA, multiplex PCR amplification (12 cycles) of the candidate MDMs *—Roche Diagnostics (Indianapolis, IN, USA) Cobas e411
Loree et al. [13]	180	Venous blood and tissue biopsies	TMB	GuardantOMNI next-generation sequencing 2.15 Mb, 500 gene panel
Arter et al. [14]	14,812	Tissue biopsies	TMB	MSK-IMPACT, Minerva panel, Samsung panel, Whole exome seq, FMI panel
Pietrantonio et al. [15]	24	Tissue biopsies	RET fusions	Minerva panel sequencing
Bartlett et al. [16]	549	Venous blood	miRNAs	xCell, TIMER, and CIBERSORT
Piha-Paul et al. [17]	18	Tissue biopsies	PI3K mutations	NP

* Abbreviations: CpG: cytosine-guanine (CpG) motifs, cfDNA: cell-free DNA, MDMs: methylated DNA markers, TMB: tumor mutation burden, RET: Rearranged during Transfection gene, miRNA: microRNA, PI3K: Phosphatidylinositol 3-kinase pathway, NP: not provided.

**Table 4:** Most frequent molecular imprints.

Researcher	n (Patients)	Most Frequent Gene/Pathway Alterations	MSI Status	Key Molecular Correlations
Chatila et al. [10]	692	*APC* (81%), *TP53* (81%), *KRAS* (42%), *FBXW7* (14%), *PIK3CA* (12%), *WNT* (85%) and *RAS* (51%)	692 MSS; 36 MSI; 4 POLE hypermutant	*APC* less frequent in lower rectal tumors; *KRAS* and *AMER1* co-occur; *TP53* mutually exclusive with *PIK3CA/KRAS*; *APC* C-terminal mutations co-occur with *KRAS* and AMER1; *PIK3CA* mutations subclonal (30%)
Gouda et al. [11]	20	*KRAS* mutation (100%)	NP	CpG sites more frequent in CRC; targeted methylation sequencing detected cfDNA in 85%
Zhu et al. [12]	35	BRAF V600E wild-type, RAS wild-type	MSS	
Loree et al. [13]	180	APC (63%), TP53 (55%), KRAS (51%), ATM (14%), BRCA2 (12%)	MSS	High concordance of MSI status between plasma and tissue
Arter et al. [14]	14,812	BRAF V600E, KRAS G12A/C/D; FGFR1 (15%), EGFR (14%), ERBB2 (7%), NTRK1 (7%), RET (7%)	38% of BRAF-mutated were MSI-H; rest NP	MSI-H group had higher TMB (56.1 ± 19.7) vs. MSS group (11 ± 18.4), *p* < 0.01
Pietrantonio et al. [15]	24	RET fusions: NCOA4-RET (12), CCDC6-RET (8), TRIM24-RET (2), TNIP1-RET (1), SNRNP70-RET (1)	MSI-H (48%), MSS (52%)	*RET* fusions more common in patients > 66 years
Bartlett et al. [16]	549	SMAD2, ACVR1B, SKP1, ACVR2B, SMAD4, ZFYVE9, ACVR2A, SP1, EP300, TGFBR2	MSS/MSI-L: 472; MSI-H: 76	41 miRNAs correlated with mutation burden; 62 with MSI; 17 with PD-L1; 3 miRNAs linked to all three plus M1 macrophage polarization
Piha-Paul et al. [17]	18	*PIK3CA* gene mutations and *PTEN* gene aberrations	NP	

Abbreviations: APC: Adenomatous Polyposis Coli gene, TP53: Tumor Protein p53 gene, KRAS: Kirsten rat sarcoma viral oncogene homolog gene, FBXW7: F-box and WD repeat domain containing 7 gene, PIK3CA: Phosphatidylinositol-4,5-bisphosphate 3-kinase catalytic subunit alpha gene, WNT: Wingless-related integration site pathway, BRAF V600E: B-Raf proto-oncogene mutation of replacement of Valine (V) by glutamic acid (E) at position 600, ATM: Ataxia-Telangiectasia Mutated, BRCA2: Breast Cancer gene 2, FGFR1: Fibroblast Growth Factor Receptor 1 gene, EGFR: Epidermal Growth Factor Receptor gene, ERBB2: Erb-B2 Receptor Tyrosine Kinase 2 gene, NTRK1: Neurotrophic Receptor Tyrosine Kinase 1 gene, RET: Rearranged during Transfection gene, NCOA4-RET: Nuclear Receptor Coactivator 4 (NCOA4) gene with the Rearranged during Transfection (RET) gene, TNIP1-RET: TNFAIP3 Interacting Protein 1 gene, SNRNP70: small nuclear ribonucleoprotein U1 subunit 70, SMAD2/4: Mothers against decapentaplegic homolog 2/4, ACVR: Activin Receptor Type, SKP1: S-phase kinase-associated protein 1 gene, ZFYVE9: Zinc finger FYVE-type containing 9 gene, EP300: E1A Binding Protein p300, TGFBR2: Transforming Growth Factor Beta Receptor 2, PIK3CA: Phosphatidylinositol 3-kinase CA mutation, *PTEN*: Phosphatase and Tensin homolog deleted on chromosome 10, MSI-L: microsatellite instability low index, exonuclease domain of the DNA polymerase epsilon (POLE) gene, NP: not provided.

**Table 5:** Agnostic biomarkers of oncological outcomes and response to CRC treatments.

Researcher	n	Agnostic Target(s)	Agnostic Biomarker(s)	Neoadjuvant Regimen(s)	Key Oncological Outcomes
Chatila et al. [10]	692	*BRAF* V600E mutations, ERBB2 amplifications, *KRAS G12C* mutations	*PIK3CA*, *NRAS*, *ATM mutations*; *CA9*; *IGF2*, *L1CAM*; immune checkpoint genes (PDCD1, CD274, CTLA4, HAVCR2, LAG3)	CRT ± CNCT; neoadjuvant INCT + CRT	KRAS mutations linked to shorter DFS in CRT–CNCT group; immune-hot MSS tumors showed favorable ICI response
Gouda et al. [11]	20	CpG methylated islands			Methylated ctDNA linked to shorter PFS (8 vs. 54 weeks, *p* = 0.027)
Zhu et al. [12]	35		13 MDMs (CNNM1, ANKRD13B, FER1L4, etc.)	FOLFOX	MDM-positive score preceded recurrence by median 106 days; rising MDM with stable CEA predicted recurrence
Loree et al. [13]	180	PD-L1, CTLA4	pTMB	durvalumab + tremelimumab	OS increased by 2.5 mo vs. BSC (HR 0.72); no PFS benefit
Arter et al. [14]	14,812	*NTRK* fusions, *RET* fusions	TMB	RTK inhibitors (pembrolizumab, dostarlimab, nivolumab + ipilimumab)	
Pietrantonio et al. [15]	24	*RET* fusions (e.g., CCDC6-RET)		RET inhibitors (RXDX-105, pembrolizumab, nivolumab)	
Bartlett et al. [16]	549	TGF-β, PD-L1 receptors	MSI status, PD-L1 status, TMB	Neutralizing antibodies, ligand traps, small-molecule inhibitors, antisense oligonucleotides	No miRNAs in any group were associated with OS
Piha-Paul et al. [17]	18	activated PI3K pathway		Buparlisib	No clinical benefit

Abbreviations: BRAF V600E: B-Raf proto-oncogene mutation of replacement of Valine (V) by glutamic acid (E) at position 600, ERBB2: Erb-B2 Receptor Tyrosine Kinase 2 gene, KRAS: Kirsten rat sarcoma viral oncogene homolog gene, PIK3CA: Phosphatidylinositol-4,5-bisphosphate 3-kinase catalytic subunit alpha gene, NRAS: neuroblastoma RAS viral oncogene homolog, ATM: Ataxia-Telangiectasia Mutated, CA9: Carbonic anhydrase 9, IGF2: insulin-like growth factor 2, L1CAM: L1 cell adhesion molecule, PDCD1 (PD-1): Programmed Cell Death Protein 1, CD274 (PD-L1): Programmed Death-Ligand 1, CTLA4: Cytotoxic T-Lymphocyte-Associated Protein 4, HAVCR2 (TIM-3): Hepatitis A Virus Cellular Receptor 2/T-cell Immunoglobulin and Mucin-domain containing-3, LAG3: Lymphocyte-Activation Gene 3, CRT: Chemoradiotherapy, CNCT: consolidative chemotherapy, INCT: induction chemotherapy, DFS: disease-free survival, CpG: Cytosine-phosphate-Guanine, ctDNA: circulating tumor DNA, MDMs: Methylation-Driven Markers, CNNM1: Cyclin and CBS Domain Divalent Metal Cation Transport Mediator 1, ANKRD13B: Ankyrin Repeat Domain 13B, FER1L4: Fer-1 Like Family Member 4, CRC: colorectal cancer, CEA: Carcinoembryonic Antigen, PD-L1: Programmed Death-Ligand 1, pTMB: Persistent Tumor Mutational Burden, OS: overall survival, BSC: Best Supportive Care, PFS: progression-free survival, RET: Rearranged during Transfection, TMB: tumor mutational burden, RTK: receptor tyrosine kinase, CCDC6: Coiled-Coil Domain Containing, TGF-β: Transforming Growth Factor beta, MSI: microsatellite instability, miRNA: microRNA, PI3K: Phosphatidylinositol 3-kinase.

## Data Availability

The data used to support the findings of this study are available from the corresponding author upon request.
